# Choroidal Changes in Diabetic Patients With Different Stages of Diabetic Retinopathy

**DOI:** 10.7759/cureus.10871

**Published:** 2020-10-09

**Authors:** Tariq Hamadneh, Saba Aftab, Nazleen Sherali, Rishwanth Vetrivel Suresh, Nicholas Tsouklidis, MeiXia An

**Affiliations:** 1 Ophthalmology, The Third Affiliated Hospital of Southern Medical University, Guangzhou, CHN; 2 Ophthalmology, California Institute of Behavioral Neurosciences & Psychology, Fairfield, USA; 3 Medicine, Hamdard College of Medicine and Dentistry, Karachi, PAK; 4 Medicine, California Institute of Behavioral Neurosciences & Psychology, Fairfield, USA; 5 Medicine, Liaquat University of Medical and Health Sciences, Jamshoro, PAK; 6 Health Care Administration, University of Cincinnati Health, Cincinnati, USA; 7 Medicine, Atlantic University School of Medicine, Gros Islet, LCA

**Keywords:** choroids, sattler's layer, haller's layer, choriocapillaris, diabetic retinopathies, diabetic, retinopathy, choroidal thickness, choroidal vascularity index, cvi

## Abstract

Diabetic retinopathy (DR) is one of the long-term microvascular complications of diabetes mellitus (DM) and is considered a leading cause of vision loss worldwide. Chronic hyperglycemia can cause microvascular abnormalities to the retina and the choroid as well. The vascular tissue of the choroid supplies blood to the outer retina, photoreceptors, and retinal pigment epithelium. It plays an important role in the metabolic exchange of the retina. Many experimental studies reported that choroidal pathology in diabetic patients might play a role in developing DR. Choroidal thickness (CT) can reflect changes in the vasculature of the choroid and can be used to assess the vascularity of the choroid itself. CT differs between healthy and diseased states of the eye as well as with the aging process. This means that thinner or thicker choroid may indicate an ocular disease. Choroidal vascularity index (CVI) is also used as a marker for choroidal vascularity assessment and indirectly measures choroidal vascularity quantitatively. Many studies have been conducted to evaluate the choroid in many different ocular diseases. However, the results regarding CT in DM, especially in patients with DR, are various as thickened, thinned, or no changes. Thus, the status of the choroid in patients with DM with or without DR remains controversial between researchers. In this systematic review, we reviewed 18 articles that were done to investigate the relationship between structural choroidal changes in diabetic patients with different stages of DR, focusing on CT, CVI, and some other parameters evaluating choroidal changes.

## Introduction and background

Diabetes mellitus (DM) is classified into type one diabetes mellitus, formerly known as insulin-dependent diabetes mellitus (IDDM), and type two diabetes mellitus, formerly known as non-insulin-dependent diabetes mellitus (NIDDM). Type one DM usually results from the destruction of pancreatic β-cells, leading to absolute insulin deficiency. Type two diabetes mellitus is characterized by insulin resistance that may or may not be accompanied by insulin deficiency. There are also other forms of DM, such as genetically mediated form secondary to endocrinopathy, drug- or chemical-induced DM [1].

Diabetic retinopathy (DR) is one of the long-term microvascular complications of DM and is believed to be a leading cause of vision loss worldwide among patients aged 25-74 years, especially in developed countries such as the United States. DR is classified clinically according to the severity of the disease into stages. In non-proliferative diabetic retinopathy (NPDR), there are changes in the intraretinal vasculature, but without newly formed extraretinal fibrovascular tissue. NPDR is also classified into mild, moderate, and severe forms according to severity. Proliferative diabetic retinopathy (PDR) is the most advanced stage of DR, which usually results after progression through the sequential stages of NPDR. PDR is characterized by the neovascularization of the retina due to chronic ischemia induced by DM. PDR is classified clinically into either early PDR or PDR with high-risk characteristics. Diabetic macular edema (DME) is characterized by swelling of the central retina. It is mainly caused by retinal vascular hyperpermeability, which can be developed in any stage of DR. DME is classified as a center-involved and non-center involved DME based on retinal subfield thickening of optical coherence tomography (OCT) [1].

The prevalence of DM is increasing globally due to the increased life expectancy and the improvement in controlling blood sugar levels. It is expected that DM will affect 642 million individuals by 2040, leading to an increased prevalence of DR as well. One-third of the global diabetic patients are estimated to have DR, and one-third of DR patients are estimated to have sight-threatening DR. There is a direct association between the duration of DM and the prevalence of DR [1].

The chronic hyperglycemia in diabetic patients results in biochemical and molecular pathway changes such as increases in inflammatory oxidative stress, advanced glycation end products, and protein kinase C pathways that ultimately cause endothelial damage and pericyte loss, which can explain the pathogenesis of DR in these patients. Over time, basement membrane thickening and selective loss of pericytes in retinal capillaries cause occlusion of these capillaries leading to decreased retinal perfusion, which ultimately leads to retinal neovascularization that develops in response to increased intraocular vascular endothelial growth factor (VEGF) secretion produced by ischemic retinal tissue. Moreover, endothelial damage can lead to the development of retinal edema due to serum leakage [1]. The chronic hyperglycemia can cause microvascular abnormalities to the choroid as well. The choroid is a vascular layer with pigmented stroma between the retina and the sclera. The choroid is supplied by the posterior ciliary arteries. The outer layer of the choroid, known as the Haller layer, contains large caliber vessels. These vessels divide into smaller caliber vessels in a layer known as the Sattler layer [1].

The vascular tissue of the choroid supplies blood to the outer retina, photoreceptors, and retinal pigment epithelium. It plays an important role in the metabolic exchange of the avascular fovea [2]. Numerous experimental studies have reported that choroidal pathology in diabetic patients may play a role in developing DR [3,4]. It was noticed that some pathological changes in the vessels of the choroid are similar to those observed in retinal vessels of DR patients [3]. The thickness of the choroid is maximum posteriorly and becomes thinner more anteriorly. The thickness of the choroid in the central macular area and at the ora serrata is 0.22 mm and 0.1 mm, respectively. In healthy volunteers with a mean age of 50, subfoveal choroidal thickness (SFCT) measured by spectral-domain optical coherence tomography (SD-OCT) is 287 μm. The thickness of the choroid differs between healthy and diseased states of the eye as well as with the aging process. This means that thinner or thicker choroid may indicate an underlying eye disease [1]. Choroidal thickness (CT) reflects changes in the choroidal vasculature, and this can be used to evaluate choroidal vascular abnormalities and its association with ocular diseases. Many studies have used CT as an indicator of choroidal and retinal blood flow [5-8]. However, there are many factors that may affect CT requiring more investigation about its effectiveness as a marker for the assessment of choroidal and retinal vascular structural characteristics.

With the recent improvement in imaging techniques, the choroid is studied and evaluated by indocyanine green angiography (ICGA) and laser doppler flowmetry [4,9,10]. More recently, the choroid is better evaluated by the enhanced depth imaging-optical coherence tomography (EDI-OCT), which can obtain cross-sectional images with high quality and resolution [11]. EDI-SD-OCT can measure CT and changes in choroidal vessels in several chorioretinal diseases [11]. Recent advances in swept-source-optical coherence tomography (SS-OCT) systems, which utilize longer wavelengths and faster scanning speeds, have helped us better visualize the choroidal vasculature, even microvascular changes due to improved tissue penetration and spatial resolution [12].

Agrawal et al. proposed the choroidal vascularity index (CVI) as a marker to assess vascular structure using EDI-OCT [13]. Using OCT, the total choroidal area (TCA), the stromal area (SA), and vascular luminal area (LA) can be measured. CVI, which is defined as the proportion of LA to TCA, is considered a more robust marker for choroidal vascularity assessment and indirectly measures choroidal vascularity quantitatively, which enables us to overcome the limitation of using CT alone and makes it less affected by physiological factors [14]. Many studies have been conducted to evaluate the choroid in many different eye diseases. However, the results regarding CT in DM, especially in patients with DR, were various as thickened, thinned, or no changes. Thus, the status of the choroid in patients with DM, with or without DR, remains controversial and needs further investigations.

This systematic review aims to review the recent literature of choroidal structural changes in diabetic patients and to see if there is a relationship between these choroidal structural changes and the severity of DR. We also want to review the different measurement variables used to assess changes in the choroid structures and how these measurement variables can help in the diagnosis, prognosis, and possible implication in the management of diabetic patients with or without DR.

## Review

Method

PubMed database was exclusively and systematically accredited for the collection of corresponding data. The first search based on using keywords, medical subject headings (MeSH) terms and MeSH subheadings, yielded 252 results. After applying inclusion and exclusion criteria (free full text, last 10 years, English language), 44 scientific articles were found. After reviewing the articles and eliminating abstract reviews and less relevant articles not specifying the outcome of interest, 18 scientific papers were included in our final review. All 18 articles met the quality specification and were peer-reviewed. 

Inclusion and exclusion criteria

All selected scientific papers were written in English and included data collected and reviewed from the last 10 years. Free full-text papers were exclusively reviewed. Many studies measured more than one parameter in patients with diabetes mellitus and DR. Papers investigating the parameters and markers related to the choroid structure were included, mainly CT and CVI. Papers excluded were not investigating changes of the choroid itself. 

Results

Table 1 contains some general information about the studies reviewed in this article. Endo et al., Wang and Tao, Gupta et al., Rewbury et al., Ohara et al., Tan et al., Hua et al., Kim et al., Sudhalkar et al., Totan et al., Gerendas et al., Lee et al., Adhi et al., Regatieri et al., and Gołębiewska et al. evaluated CT measurements and some of these studies also evaluated CVI in diabetic patients with and without DR as described in Table 2 [14-28]. Kim et al. only investigated for CVI in these patients [29]. Dodo et al. and Nesper et al. used choroidal parameters other than CT and CVI to evaluate choroidal changes [30,31].

**Table 1:** General descriptive details of the studies from the review. BCVA: best corrected visual acuity, CSME: clinically significant macular edema, DM: diabetes mellitus, DME: diabetic macular edema, DR: diabetic retinopathy, EDI-OCT: enhanced depth imaging optical coherence tomography, HD-OCT: high-definition optical coherence tomography, ME: macular edema, mmNPDR: mild-to-moderate non-proliferative diabetic retinopathy, msNPDR: moderate-to-severe non-proliferative diabetic retinopathy, NPDR: non-proliferative diabetic retinopathy, OCTA: optical coherence tomography angiography, PDR: proliferative diabetic retinopathy, PRP: pan-retinal photocoagulation, SMD: serous macular detachment, SS-OCT: swept-source optical coherence tomography.

Study	Location	Study Period	Samples	Method
Endo et al. [15]	Japan	December 2013-April 2018	318 eyes of 159 DM patients and age-matched 100 eyes of 79 healthy controls. Cases have no ocular treatment history.	EDI-OCT
Wang and Tao [16]	China	2019	104 eyes, divided into four groups: healthy controls (n=38), DM without DR eyes (n=22), PRP-untreated NPDR eyes (n=24), PRP-untreated PDR eyes (n=20).	EDI-OCT
Gupta et al. [17]	South India	September 1, 2015-December 31, 2016	82 eyes of 52 patients with treatment-naive DME with varying grades of DR and 86 eyes of 43 healthy control patients.	EDI-OCT
Rewbury et al. [18]	Oxford, UK	January 2012-February 2013	145 eyes from 95 patients with untreated type two DM and moderate-to-severe visual loss were included. Eyes were divided into two groups based on the presence or absence of foveal involving DME and classified according to retinopathy grade: R1 (mild NPDR) (n=87), R2 (msNPDR) (n=37), and R3 (PDR) (n=21).	EDI-OCT
Ohara et al. [19]	Japan	August 2014-March 2015	Healthy subjects (n=20), diabetic patients without DR (n=14), mmNPDR (n=16), severe NPDR (n=11) and PDR without PRP (n=18) [total is 79 people] were enrolled. For eyes with DR, only treatment-naive eyes were included. 32 eyes [19 eyes with PDR and 13 eyes with severe NPDR] for which PRP was necessary were also analyzed.	SS-OCT
Tan et al. [20]	Singapore	2015	38 eyes of 19 age and gender-matched healthy controls and 38 eyes of 19 patients with DM were analyzed.	EDI-OCT
Hua et al. [21]	China	2013	The study included 80 eyes from 40 patients. Group one consisted of 62 eyes from 31 DR cases, which were qualitatively assessed. This group included 13 eyes with mild NPDR, 11 with moderate NPDR, 15 with severe NPDR, and 23 with PDR. Group two consisted of 18 DME eyes from nine NPDR cases, which were quantitatively assessed for choroids. In each case, one eye had SMD and was placed in the SMD subgroup while the other eye did not have SMD and was placed in the non-SMD sub-group. Three of the nine cases in Group two had a history of biocular PRP and were designated as the PRP-treated sub-group. The other six cases were placed in the non-PRP-treated sub-group.	EDI-OCT
Kim et al. [14]	Seoul, Republic of Korea	December 2016-April 2017	The study included 185 eyes of patients with a confirmed diagnosis of type two DM and 45 eyes of healthy controls.	SS-OCT
Sudhalkar et al. [22]	India	September 2012-July 2013	The study group had 227 eyes of 125 subjects with type two DM. Out of 227 eyes, 74 eyes did not have any evidence of DR, 89 eyes of NPDR, 33 eyes had treatment naïve PDR, and 31 eyes with PRP treated PDR. The control group eventually consisted of 197 eyes of 110 healthy subjects.	EDI-OCT
Totan et al. [23]	Turkey	December 2013-August 2014	A total of 34 patients who had type two DM with treatment naïve DME and 34 sex-matched healthy subjects were included. Only one eye from each subject was included for analysis. If both eyes qualified, the eye with worse BCVA was selected.	Cirrus HD-OCT
Gerendas et al. [24]	Vienna, Austria	2013	284 treatment-naïve eyes of 142 patients with CSME and 20 controls. 27 patients (19%) were excluded. Accordingly, data from 115 patients (81%) were processed by an automated analysis.	SD-OCT
Lee et al. [25]	Korea	October 2011-June 2012	203 eyes of 203 diabetic participants and 48 eyes of 48 healthy controls. The study population included 59 eyes with no diabetic change, 56 eyes with mmNPDR, 40 eyes with severe NPDR, and 48 eyes with PDR. Only naive eyes of various DR grades were included.	EDI-OCT
Adhi et al. [26]	USA	February 1, 2010-June 30, 2012	33 eyes of 33 patients with DR and 24 eyes of 24 controls. Patients were classified into NPDR without ME (nine eyes), PDR without ME (10 eyes), and DME (14 eyes).	SD-OCT
Regatieri et al. [27]	USA	December 2009-June 2010	49 eyes of 49 type two DM patients and 24 eyes of 24 normal patients. The patients with DM were classified into three groups, 11 patients (11 eyes) with mmNPDR and no ME (NPDR group), 18 patients (18 eyes) with mmNPDR and DME (DME group), and 20 patients (20 eyes) with treated PDR and no DME (treated PDR group).	Cirrus HD-OCT
Gołębiewska et al. [28]	Poland	2017	64 right eyes of 64 subjects with type one DM and 45 right eyes of 45 age-matched healthy volunteers (control group) were enrolled in this study. The mean age of the subjects and controls was 15.3 and 14.6, respectively.	SD-OCT
Kim et al. [29]	Seoul, Republic of Korea	December 2016-December 2017	A total of 174 eyes (132 eyes of 81 patients with type two DM and 42 eyes of 28 healthy controls). We divided eyes into six groups: healthy controls (n=42), no DR (n=30), mild NPDR (n=22), moderate NPDR (n=23), severe NPDR (n=42), and PDR (n=15).	SS-OCT and OCTA
Dodo et al. [30]	Japan	February 2015-August 2016	108 eyes of 66 consecutive patients with DM were evaluated.	OCTA
Nesper et al. [31]	Chicago, Illinois, USA	June 2015-July 2016	137 eyes of 86 patients with different stages of DR and 44 eyes of 26 healthy age-matched controls.	OCTA

**Table 2:** Results of the main choroidal parameters of the studies from the review. CA: choroidal area, CC: choriocapillaris, CCT: central choroidal thickness, CSME: clinically significant macular edema, CT: choroidal thickness, CVI: choroidal vascular index, DME: diabetic macular edema, DT: systemic diabetic treatment, LA: luminal area, L/C ratio: luminal-to-choroidal ratio, ME: macular edema, mmNPDR: mild-to-moderate non-proliferative diabetic retinopathy, msNPDR: moderate-to-severe non-proliferative diabetic retinopathy, NPDR: non-proliferative diabetic retinopathy, OCTA: optical coherence tomography angiography, PDR: proliferative diabetic retinopathy, PRP: pan-retinal photocoagulation, SA: stromal area, SFCT: subfoveal choroidal thickness, SMD: serous macular detachment, TCA: total choroidal area.

Study	Groups	Choroidal Part	Thickness Measurement (μm)	CVI	Conclusion
Endo et al. [15]	Healthy control	Total CCT	254±83		The total and outer CCT layers of diabetic eyes were significantly thickened in the DME+DT− as compared with the DME−DT+ group. The total CCT layer and the outer choroidal layer thickness were significantly thicker in the DME+ than in the DME− group in all DME cases examined.
	DME+		283±88		
	DME−		251±70		
	DME+DT+		274±88		
	DME−DT+		247±66		
	DME+DT−		290±84		
	DME−DT−		258±75		
	Healthy control	Outer CCT	195±75		
	DME+		222±83		
	DME−		193±63		
	DME+DT+		214±83		
	DME−DT+		189±58		
	DME+DT−		228±77		
	DME−DT−		201±70		
Wang and Tao [16]	DM	SFCT	213.21±19.02	0.63±0.04	Eyes of patients with DM showed that the L/C ratio (equals to CVI) decreased compared with normal controls. The SFCT increased, but the L/C ratio significantly decreased with worsening of DR compared with DM with no DR, and normal eyes.
	Healthy control		212.63±11.99	0.68±0.06	
	DM without DR		194.18±5.68	0.65±0.03	
	PRP-untreated NPDR		217.29±14.07	0.63±0.05	
	PRP-untreated PDR		229.25±13.89	0.61±0.04	
Gupta et al. [17]	Healthy control	SFCT	284.53±56.45	67.51±2.86	SFCT was significantly increased in eyes with DME as compared to the controls and showed an ascending trend with worsening of DR, though this difference was not statistically significant. CVI was significantly decreased in DME with DR eyes as compared to controls. CVI was also significantly decreased with worsening DR.
	Mild NPDR		304.33±40.39	66.38±0.3	
	Moderate NPDR		327.81±47.39	65.28±0.37	
	Severe NPDR		357.72±62.65	63.50±0.47	
	PDR		334.59±47.4	61.27±0.9	
	DME		334.47±51.81	63.89±1.89	
Rewbury et al. [18]	Mild NPDR	Mean SFCT	217.7±62		SFCT increased with the severity of DR, especially in the PDR group. DME was associated with a non-statistically significant increase in CT compared with eyes without DME.
	msNPDR		221.7±62		
	PDR		242.1±48		
	DME−		209.3±61		
	DME+		225.4±60		
Ohara et al. [19]	Before PRP	SFCT	268.4±102.9		CT significantly decreased after PRP, which continued for at least six months after treatment. CT of severe NPDR and PDR was significantly thicker than that of mild-to-moderate NPDR.
	One month after PRP		253.4±103.1		
	Three months after PRP		253.8±107.1		
	Six months after PRP		252.9±110.5		
	Healthy control		243±71.4		
	DM without DR		251.3±61.9		
	mmNPDR		227.1±71.3		
	Severe NPDR		323.1±66.0		
	PDR		301.7±80.8		
	before PRP	Central Field CT	268.6±104.5		
	One month after PRP		254.5±105.3		
	Three months after PRP		254.2±108.2		
	Six months after PRP		248.1±101.8		
	Healthy control		248.3±70.7		
	DM without DR		250.2±55.4		
	mmNPDR		230.0±70.3		
	Severe NPDR		323.2±61.3		
	PDR		307.3±84.1		
Tan et al. [20]	Healthy control	Average Choroidal (subfoveal, nasal, and temporal)	180.4±70.50	67.20±0.16	Eyes of patients with DM showed significantly decreased CVI with no corresponding change in CT compared to controls. However, there was a significant decrease in CVI and an increase in TCA, LA, SA, and average CT in DR patients compared with DM without DR.
	DM group		168.37±52.07	65.10±0.20	
	DM without DR		157.24±48.29	65.30±0.21	
	DR group		193.68±53.65	64.20±0.16	
Hua et al. [21]	DME+ SMD−	SFCT	276		In group two, both the SFCT and CA of eyes with DME and SMD were significantly greater than those in the other eyes. The CA in PRP treated cases was also greater than that in non-PRP treated cases.
	DME+ SMD+		364		
	Non-PRP treated		288		
	PRP-treated		365		
Kim et al. [14]	Healthy controls	SFCT	320±77.92	69.08±2.29	The eyes of patients with DM, even without DR, exhibited a significantly lower CVI than those of healthy controls. Notably, the PDR group exhibited a significantly lower mean CVI relative to the other DR stages. Eyes of diabetic patients exhibited a lower SFCT than the eyes of healthy controls. Among the eyes of diabetic patients, the lowest CT values were observed in the PDR group.
	DM without DR		258.13±89.02	67.07±3.71	
	mmNPDR		310.22±72.41	66.28±2.70	
	Severe NPDR		304.53±69.26	66.2±2.56	
	PDR		258.75±73.29	63.48±2.89	
	PRP-treated DR		276.29±79.51	65.38±3.15	
	CSME		312.58±89.59	66.28±2.85	
Sudhalkar et al. [22]	Healthy controls	Mean SFCT	281.7±47.7		Control eyes had greater SFCT compared to subjects with DM, with and without retinopathy. The thinning progressed with increasing severity of DR. SFCT in eyes with ME was not significantly different from eyes without ME.
	DM without DR		261.71±51.8		
	DM with any form of DR		252.8±55.6		
	NPDR		248.0±56.3		
	PDR		243.9±56.2		
	PRP-treated DR		258.4±48.3		
	Non-PRP-treated DR		251.78±56.9		
	DME−		246.805±55.61		
	DME+		256.629±55.24		
Totan et al. [23]	Healthy controls	SFCT	321.4±36.5		Both pulsatile choroidal blood flow and CT were decreased in patients with DME.
	DME		273.5±30.2		
Gerendas et al. [24]	Healthy controls	Total CT in the 6-mm region on the foveal grid	190±23		Total CT in the 6-mm region on the foveal grid is significantly reduced in DME and non-edematous fellow eyes of patients compared with healthy control eyes. There was no statistically significant difference in overall CT between patients’ study eyes with DME and their fellow eyes without DME.
	Non-edematous fellow eyes		177±20		
	DME		175±23		
Lee et al. [25]	Healthy controls	SFCT	228.5±38.9		The CT of subfoveal regions was significantly decreased in DR patients compared with controls. The proliferative changes or presence of ME did not result in additional choroidal thinning.
	No diabetic change		219.1±47.8		
	mmNPDR		158.9±56.3		
	Severe NPDR		161.2±38.5		
	PDR		157.4±45.7		
	DME+		164.1±63.0		
	DME−		157.2±71.1		
Adhi et al. [26]	Healthy controls	SFCT	276.4±13.4		Choroidal morphological features are altered in patients with moderate to severe DR. The SFCT and the subfoveal medium choroidal vessel layer and CC layer thicknesses are significantly reduced in patients with DR, PDR, and DME compared to controls.
	NPDR		252.9±20.27		
	PDR		209.6±12.42		
	DME		211.6±17.05		
Regatieri et al. [27]	Normal	Mean SFCT	232.3±15.2		There is a significant decrease in the CT in patients with DME or treated PDR compared to normal subjects. No significant difference between normal subjects and the NPDR group was observed. Between DME and treated PDR groups, there was no significant difference.
	NPDR		222±21.6		
	DME		169.5±14.7		
	PDR		162.7±7.0		
Gołębiewska et al. [28]	Diabetic	SFCT	355.65		CT remains unchanged in children with Type one DM. There was no significant difference between subjects and controls in the CT in the fovea, nasal, temporal, superior, and inferior quadrants of the macula. However, regardless of the prevalence of Type one DM in the studied children, CT was significantly thicker in girls than in boys, except for the superior quadrant.
				Non-diabetic		327.98	
	Kim et al. [29]		Healthy controls						69.21±2.24	CVI correlated negatively with worsening DR severity, P-value= 0.009.
			DM without DR				67.06±3.98			
Mild NPDR				66.60±3.03						
Moderate NPDR				66.18±3.04						
Severe NPDR				66.15±2.63						
PDR				63.10±3.45						
Dodo et al. [30]					On the ~10-μm and ~29-μm-thick CC slab images, the areas of flow void increased gradually according to the DR severity, and eyes with severe NPDR and PDR had significantly larger areas of flow void and larger non-flow areas than those with no apparent retinopathy. In 12 eyes with ischemic maculopathy, the CC layer beneath the disrupted ellipsoid zone of the photoreceptor (EZ) had greater areas of flow void than did the area beneath an intact EZ.
Nesper et al. [31]					Retinal and CC vascular nonperfusion in OCTA increased significantly with disease severity in eyes with DR.

In Wang and Tao, for TCA and SA, the pairwise comparisons showed 0.81±0.06 mm^2^; 0.28±0.03 mm^2^, 0.86±0.09 mm^2^; 0.31±0.05 mm^2^, 0.90±0.08 mm^2^; 0.34±0.03 mm^2^ for DM without DR eyes, pan-retinal photocoagulation (PRP)-untreated NPDR eyes, and PRP-untreated PDR eyes, respectively [16]. 

In Tan et al., TCA, LA, and SA values were 0.81±0.25 mm^2^, 0.54±0.16 mm^2^, and 0.27±0.09 mm^2^ in controls and 0.83±0.28 mm^2^, 0.54±0.18 mm^2^, and 0.29±0.10 mm^2^ in DM group. The same values were 0.75±0.28 mm^2^, 0.49±0.18 mm^2^, and 0.26±0.10 mm^2^ in DM with no DR and 0.97±0.24 mm^2^, 0.62±0.15 mm^2^, and 0.34±0.09 mm^2^ in DR group [20].

In Hua et al., in group two (DME group), choroidal area (CA) was 906.246 μm^2^, 798,066 μm^2^, 947,448 μm^2^, and 777,474 μm^2^ in eyes with DME and serous macular detachment (SMD), eyes with DME without SMD, PRP treated, and non-PRP treated cases, respectively [21].

Totan et al. concluded that the mean ocular pulse amplitude (OPA) values were 2.58±0.96, 3.52±1.03 in patients with DME and controls, respectively [23].

In Adhi et al., large vessel choroidal layer thickness was 224.0±13.5 μm, 206.6±18.01 μm, 169.2±12.43 μm, and 173.7±14.63 μm in healthy eyes, NPDR, PDR, and DME eyes, respectively. Medium choroidal vessel layer and choriocapillaris (CC) layer thickness was 52.5±1.8 μm, 46.3±3.6 μm, 40.4±2.96 μm, and 37.93±3.3 μm in the same groups, respectively [26].

In Gołębiewska et al., CT in the fovea, nasal, temporal, superior, and inferior quadrants of the macula was 355.65 μm, 282.32 μm, 338.88 μm, 342.40 μm, and 352.35 μm in diabetic and 327.98 μm, 267.16 μm, 324.07 μm, 328.70 μm, and 354.56 μm in the non-diabetic group, respectively [28].

Dodo et al. found that on the ~10-μm and ~29-μm-thick CC slab images, according to the DR severity, the areas of flow void increased gradually. In 12 eyes with ischemic maculopathy, the CC layer beneath the intact ellipsoid zone of the photoreceptor (EZ) and beneath the disrupted EZ had area of flow void as 2.00±1.10%, 6.28±3.34%, respectively [30].

In Nesper et al., the percent area of nonperfusion (PAN) measured in the CC was 2.53±0.69%, 3.39±1.37%, 3.37±1.08%, and 4.40±1.68% in healthy controls, DM without DR, NPDR, and PDR, respectively. The mean adjusted flow index (AFI) in the CC was 0.393±0.020, 0.392±0.022, 0.382±0.026, and 0.380±0.027 in the same groups, respectively [31].

Limitations

1. Several studies did not adjust for physiologic, systemic, and local ocular variables that can be considered as confounders and affect the results of CT. These factors include blood pressure, age, axial length, refractive error, anterior chamber depth, diurnal variation, race, duration of DM, different kinds of DM medications, exposure to PRP, intravitreal anti-VEGF, and steroids.

2. Several studies also had small sample sizes, which can reduce the strength of statistical analysis and affect the results.

3. Only free full-text articles available on the PubMed database were used.

Discussion

Studies Showing Increasing CT in DM Patients or With Worsening DR

Endo et al. found that the total central choroidal thickness (CCT) layer in the DME positive (DME+) was significantly thicker than the DME negative (DME-) group (P<0.05). The outer CCT layer in DME+ was significantly thicker than the DME- group (P<0.05). The total and outer CCT layer in DME+ without systemic diabetic treatment (DME+DT-) was significantly thicker than the DME- with systemic diabetic treatment (DME-DT+) group (P<0.05). In contrast, between the groups, there was no significant difference in the inner choroidal layer. In stage of DR, the inner CCT layer was significantly thicker in severe NPDR compared to the control group (P<0.05) [15].

In Wang and Tao, the luminal-to-choroidal area (L/C ratio) values, which are equal to CVI, were significantly decreased in DM compared to the healthy group (P<0.001). But there were no statistically significant differences in SFCT between the two groups (P=0.849). The SFCT values were significantly lower in DM without DR eyes, followed by PRP-untreated NPDR eyes and PRP-untreated PDR eyes. For TCA and SA, the pairwise comparisons showed that DM with no DR eyes were significantly lower compared with PRP-untreated NPDR eyes and PRP-untreated PDR eyes (P<0.001). Relative to the eyes of DM without DR, the L/C ratio values for eyes of PRP-untreated NPDR and PRP-untreated PDR patients were significantly lower (P=0.019) [16].

Gupta et al. found that SFCT significantly increased in eyes with DME as compared to controls (P<0.001) and showed an increasing thickness with worsening of DR, although this difference was not statistically significant (P=0.09). CVI was significantly decreased in DME with DR eyes as compared to controls (P<0.001). CVI was also significantly decreased with worsening DR (P<0.001). Patients with hypertension were significantly found to have thinner SFCT when compared with non-hypertensive patients (319.0±54.6 μm vs. 354.0±48.8μm (P=0.01)). The presence of hypertension did not affect CVI [17].

In Rewbury et al., there was a statistically significant increase in CT in PDR when compared with mild NPDR group (P=0.027). The increase in SFCT in the moderate-to-severe non-proliferative diabetic retinopathy (msNPDR) group compared with the mild NPDR group was not statistically significant (P=0.17). Compared with eyes without DME, DME was associated with a non-statistically significant increase in CT (P=0.13) [18].

In Ohara et al., the central field CT was significantly decreased at one, three, and six months after PRP (P<0.0001). SFCT was significantly decreased at one, three, and six months after treatment (P=0.0002). The central field CT of severe NPDR was significantly thicker than that of normal and mild-to-moderate non-proliferative diabetic retinopathy (mmNPDR) (P=0.0455 and 0.0099, respectively). Moreover, the central field CT of PDR was significantly thicker than mmNPDR (P=0.0169). In addition, SFCT of the PDR group was significantly thicker than that of mmNPDR group; SFCT of severe NPDR group was significantly thicker than that of normal and mmNPDR groups. The results of SFCT and central field CT were extremely similar [19].

In Tan et al., the results showed that there were no significant differences between patients with DM and controls in TCA (P=0.78), LA (P=0.90), SA (P=0.33), and average CT (P=0.40). However, there was a significantly lower CVI in patients with DM as compared to controls (P<0.0001). However, the results showed a significant increase in TCA (P=0.026), LA (P=0.036), SA (P=0.01), and average CT (P=0.05) in DR patients compared with DM without DR. Moreover, there was a significantly lower CVI in patients with DR as compared to DM without DR (P=0.035) [20].

In Hua et al., in group one (DR cases), choroidal abnormalities that were evident using ICGA but not fundus fluorescein angiography (FFA) included early hypo-fluorescent spots, late hyperfluorescent spots, and late choroidal nonperfusion regions in 75.81%, 59.68%, and 51.61% of the DR eyes, respectively. In particular, a significant difference between PDR in 17 of 23 eyes (73.91%) and non-PDR in 16 of 39 eyes (41.03%) was observed in late choroidal non-perfusion regions. In group two (DME group), both the SFCT and choroidal area (CA) of eyes with DME and serous macular detachment (SMD) were significantly greater than those in the other eyes. The CA in PRP treated cases was also greater than that in non-PRP treated cases [21].

The previous studies showed an increase in CT as DR worsens. This can be explained by the increased secretion of intraocular VEGF. DM is known to cause microvascular abnormalities, which can lead to ischemia and hypoxia in the choroidal vasculature and adjacent retinal tissue, which in turn leads to increased secretion of VEGF, a known cytokine that mediates vascular hyper-permeability and fluid leakage leading to increased CT. However, in many studies, we found that CT decreases in DM patients with no retinopathy and during the early stages of DR. This can be explained by decreasing capillary perfusion and ischemia of the choroid in the early stages, leading to a decreased vascular layer of the choroid and CT. However, with the progression of DR, hypoxia leads to more VEGF secretion leading to neovascularization, vascular hyper-permeability, fluid leakage, increased blood flow, and increased CT. These findings suggest that changes in choroidal vasculature could be the primary event in the pathogenesis of DM, even without clinical evidence of DR.

Studies Showing Decreasing CT in DM Patients or With Worsening DR

Kim et al. concluded that the eyes of DM patients exhibited a significantly lower mean CVI (P<0.001) relative to healthy controls. Notably, the PDR group exhibited a significantly lower mean CVI relative to controls (P<0.001), DM group without DR (P=0.029), and mmNPDR group (P=0.015). In comparison with other DR groups, no significant change in CVI was observed in the clinically significant macular edema (CSME) group. Eyes of DM patients exhibited a lower SFCT than the eyes of healthy controls. The lowest CT values among the eyes of DM patients were observed in the PDR group. In the DM group without DR, SFCT decreased significantly than mmNPDR group and severe NPDR group (P=0.009 and P=0.018, respectively) [14]. 

In Sudhalkar et al., a statistically significant subfoveal choroidal thinning was observed in eyes with DR when compared to subjects with DM without DR (P<0.001) and age‑matched healthy eyes (P<0.011). There was no significant difference in SFCT between eyes with DR, who had undergone PRP and eyes that had not undergone PRP (P=0.23). SFCT decreased with increasing severity of DR. As compared to those with NPDR, patients with PDR had statistically significantly thinner subfoveal choroids (P=0.021). In eyes with macular edema (ME), the mean SFCT was not significantly different from eyes without ME (P=0.196) [22]. 

In Totan et al., mean OPA values in patients with DME were statistically decreased compared with controls (P<0.001). Mean SFCT, nasal and temporal CT values were significantly decreased in DME eyes compared to controls (P<0.001) [23]. 

In Gerendas et al., total CT in the 6-mm region on the foveal grid is significantly reduced in DME (P=0.0016) and non-edematous fellow eyes (P=0.009) of patients compared with healthy controls. Overall, CT between patients’ study eyes with DME and their fellow eyes without DME had no statistically significant difference. Choroidal thinning in this study also equally affected fellow eyes without ME, suggesting a systemic pathophysiologic mechanism unrelated to the presence of retinal disease [24].

In Lee et al., SFCT was thickest among the controls. Between the no diabetic change group and the controls, there was no significant difference (P=0.846); however, a significant decrease in CT was observed in mmNPDR, severe NPDR, and PDR groups (P=0.005, P<0.001, and P< 0.001, respectively) compared with controls. There were no significant differences among mmNPDR, severe NPDR, and PDR groups (P>0.05). Eyes exhibiting ME had no significant difference in CT compared with eyes having normal macular contours [25].

In Adhi et al., the choroidoscleral interface had an irregular contour in eight of nine eyes with NPDR (89%), nine of 10 eyes with PDR (90%), and 13 of 14 eyes with DME (93%) compared with 0 of 24 controls. Mean SFCT and mean combined subfoveal medium choroidal vessel layer and CC layer thickness were significantly reduced in eyes with DR compared with controls. The same significant reduction was seen in PDR and DME groups compared to controls (P<0.05). The maximum CT was subfoveal in 22% of eyes with NPDR, 20% of eyes with PDR, and 14% of eyes with DME compared with 96% of eyes in controls. Focal thinning of the choroid with respect to the mean CT measurements at the corresponding locations in healthy eyes was observed in 0%, 56%, 40%, and 86% of eyes in controls, NPDR, PDR, and DME groups, respectively [26].

In Regatieri et al., there was a statistically significant decrease in CT of DME and treated PDR group compared to normal subjects (P<0.001). No significant difference was observed between normal subjects vs. NPDR group, and DME vs. treated PDR groups (P>0.05) [27].

In the previous section, choroidal thinning associated with DR progression might be explained by CC loss or vascular constriction secondary to choroidal hypoxia leading to decreased CT.

Studies Showing No Change in CT in DM Patients or With Worsening DR

In Gołębiewska et al., CT in the fovea, nasal, temporal, superior, and inferior quadrants of the macula did not differ statistically between the study groups: P=0.134, P=0.270, P=0.691, P=0.504, and P=0.862, respectively. There were no significant correlations between CT vs. hemoglobin A1C (HbA1C) level and duration of diabetes (P=0.197 and P=0.272, respectively). However, the CT was significantly thicker in girls than in boys, except for the superior quadrant, regardless of the prevalence of type one DM in the studied children. This difference could be due to different hormonal exposure between males and females because estrogens and progestins can have vascular effects that can affect choroidal blood flow [28]. In the previous three sections, we saw a discrepancy (increased, decreased, no change) in CT in diabetic patients with and without DR compared to healthy controls. This discrepancy can be explained by the following points. First, many studies investigated only the central choroid, and the parafoveal CT may show different results among the same studies. Second, many studies ignored considering systemic treatment for DM, and the different medications used in cases can affect CT differently. Third, many studies did not consider systemic confounding factors such as blood pressure, serum lipids, which are supposed to be higher in DM patients, which could affect the CT; for example, in Gupta et al., patients with hypertension were found to have significantly thinner SFCT when compared with non-hypertensive patients [16]. Fourth, CT measurement is different in some studies, which can affect the results obtained. Fifth, physiologic variables such as age, refractive errors, diurnal variation, axial length, races, sex, anterior chamber depth, duration of DM, smoking, and HbA1C can affect CT measurements, which were not adjusted for in many studies. Sixth, some studies have a small sample size, which decreases the statistical strength of the analysis. Seventh, different classification of DR subgroups and the inclusion of patients with type one DM in some studies can affect CT differently than in type two DM. Eighth, many studies did not include treatment naïve eyes which were treated by PRP or intravitreal anti-VEGF or steroids, which can affect CT. Moreover, the time of measuring CT after PRP or ocular injection therapy can affect CT differently. Based on these factors, we can say that CT is not a robust tool for assessing DR progression because it can be affected by many physiologic, systemic, and local factors. Moreover, CT doesn’t tell us about which part of the choroid is affected at the microscopic level (stroma, vascular layer, or fluid).

Studies Investigating Other Choroidal Parameters but Not CT in DM Patients With and Without DR

Kim et al. found that CVI negatively correlated with worsening DR severity (P=0.009) [29]. 

In Dodo et al., on the ~10-μm-thick CC slab images, according to the DR severity, the areas of flow void increased gradually, and eyes with moderate NPDR, severe NPDR, and PDR had significantly larger areas of flow void than those with no apparent retinopathy (P=0.032, P=0.009, and P=0.002, respectively). On the ~29-μm-thick CC slab images, eyes with severe NPDR and PDR had greater areas of flow void than those with no apparent retinopathy. Eyes with severe NPDR and PDR had larger non-flow areas than those with no apparent retinopathy. In 12 eyes with ischemic maculopathy, the CC layer beneath the intact ellipsoid zone of the photoreceptor (EZ) had significantly smaller areas of flow void than did the area beneath the disrupted EZ (P<0.001) [30].

Nesper et al. concluded that the percent area of nonperfusion (PAN) measured in the CC increased significantly with increasing severity of DR (P<0.01). The mean adjusted flow index (AFI) in the CC was correlated significantly negatively with DR severity (P<0.01) [31].

The results of this section confirm that choroidal vascular abnormalities are evident in DM patients and increase with worsening DR suggesting that choroidal vascular abnormalities might be an underlying pathologic process leading to DR.

CT in DME Patients

In this review, we found a discrepancy in CT measurements in DME patients in different studies (increased vs. decreased). Possible reasons for these different results include different inclusion criteria for DME patients, especially regarding different stages of DR in these patients. It’s expected that if DME patients have a more severe stage of DR, such as PDR, they are highly expected to have different CT than those in earlier stages of DR. Moreover, systemic treatment or different medications used for treating DM could have an impact on CT in these patients. VEGF is an important cytokine that mediates vascular hyper-permeability, so an increase in VEGF levels, which is expected in an advanced stage of DR and DME, can affect CT in these patients. So it is important to consider if PRP, intravitreal anti-VEGF, and steroid injection have been used or not because they can have a significant impact on CT in these patients by reducing VEGF levels and possibly decreasing CT. Moreover, the time of measuring CT after these therapies can also affect the results. For example, it is expected that early after laser therapy, CT increases due to effusion of the choroid as a result of blood vessel injuries and due to shifting of blood flow to the central choroidal area after peripheral retinal exposure to thermal injury. All the samples investigated in the previous studies should be adjusted for these factors to have a better understanding of CT in DME patients.

For those studies with no significant change or decreasing CT in DME, this could be explained by CT being more affected by DR. For those studies with increasing CT in DME, this could be due to increased VEGF with a resultant increase in neovascularization, vascular hyperpermeability, fluid leakage and blood flow leading to increased CT.

CVI in Diabetic Patients With and Without DR or ME

In Gupta et al., we found that hypertensive patients had thinner choroid compared to non-hypertensive patients in the diabetic group. However, CVI was not affected by hypertension [17]. Moreover, in almost all studies mentioned in this article that investigated CVI, there was a significant decrease in CVI with worsening DR. This consensus reflects the stability of CVI compared with CT and makes it a more robust tool in assessing DR progression and the severity of the underlying disease. CVI may represent choroidal vascular changes, which can be a valuable tool even in DM patients with no apparent DR. However, CT can reflect secondary changes of edema and leakage that can be seen due to increased VEGF secretion. Because CVI is a ratio, it is less likely to be affected by the factors affecting CT, making it a better tool to be used in the evaluation of choroidopathy. 

Recommendations

1. In future studies, inclusion of a larger sample size is required to confirm the results. 

2. It is really important to adjust the study population for the factors that can affect CT to better understand the relationship between CT and the worsening of DR.

3. Using tools or variables that are less affected by systemic and physiologic factors, such as CVI, could be more beneficial in assessing the prognosis and progression of DR.

## Conclusions

The choroid has an important contribution to the blood supply of the retina; therefore, assessment of different variables of the choroid can help in the evaluation and assessment of DR progression, which helps in the early diagnosis and treatment. The discrepancy seen in the results of various studies shows that CT does not correlate well with worsening DR or DME (can be increased or decreased) and can’t be relied upon to assess the progression and severity of DR. This discrepancy is most likely because CT can be affected by various physiologic, systemic and local factors such as blood pressure, age, axial length, refractive error, anterior chamber depth, diurnal variation, race, sex, duration of DM, different kinds of DM treatments, exposure to PRP and intravitreal anti-VEGF or steroids. Some studies also have small sample sizes, which can reduce the strength of statistical analysis and affect the results. 

CVI decreased significantly with worsening DR in almost all the studies which investigated it. CVI, which is defined as the proportion of LA to TCA, is considered a more robust marker for choroidal vascularity assessment and indirectly measures choroidal vascularity quantitatively, which enables us to overcome the limitation of using CT alone and makes it less affected by physiological factors. We postulate that CVI can be used to assess the progression of DR more accurately than CT. The results of this article affirm that choroidal vascular anomalies are apparent in DM patients and increment with worsening DR suggesting that choroidal vascular abnormalities may be a fundamental pathologic process driving to DR.
